# The *C*. *elegans* Chp/Wrch Ortholog CHW-1 Contributes to LIN-18/Ryk and LIN-17/Frizzled Signaling in Cell Polarity

**DOI:** 10.1371/journal.pone.0133226

**Published:** 2015-07-24

**Authors:** Ambrose R. Kidd, Vanessa Muñiz-Medina, Channing J. Der, Adrienne D. Cox, David J. Reiner

**Affiliations:** 1 Lineberger Comprehensive Cancer Center, University of North Carolina, Chapel Hill, North Carolina, United States of America; 2 Department of Pharmacology, University of North Carolina, Chapel Hill, North Carolina, United States of America; 3 Department of Radiation Oncology, University of North Carolina, Chapel Hill, North Carolina, United States of America; 4 Center for Translational Cancer Research, Institute of Biosciences and Technology, Texas A&M Health Science Center and College of Medicine, Houston, Texas, 77030, United States of America; Institut Pasteur, FRANCE

## Abstract

Wnt signaling controls various aspects of developmental and cell biology, as well as contributing to certain cancers. Expression of the human Rho family small GTPase Wrch/RhoU is regulated by Wnt signaling, and Wrch and its paralog Chp/RhoV are both implicated in oncogenic transformation and regulation of cytoskeletal dynamics. We performed developmental genetic analysis of the single *Caenorhabditis elegans* ortholog of Chp and Wrch, CHW-1. Using a transgenic assay of the distal tip cell migration, we found that wild-type CHW-1 is likely to be partially constitutively active and that we can alter ectopic CHW-1-dependent migration phenotypes with mutations predicted to increase or decrease intrinsic GTP hydrolysis rate. The vulval P7.p polarity decision balances multiple antagonistic Wnt signals, and also uses different types of Wnt signaling. Previously described cooperative Wnt receptors LIN-17/Frizzled and LIN-18/Ryk orient P7.p posteriorly, with LIN-17/Fz contributing approximately two-thirds of polarizing activity. CHW-1 deletion appears to equalize the contributions of these two receptors. We hypothesize that CHW-1 increases LIN-17/Fz activity at the expense of LIN-18/Ryk, thus making the contribution of these signals unequal. For P7.p to polarize correctly and form a proper vulva, LIN-17/Fz and LIN-18/Ryk antagonize other Wnt transmembrane systems VANG-1/VanGogh and CAM-1/Ror. Our genetic data suggest that LIN-17/Fz represses both VANG-1/VanGogh and CAM-1/Ror, while LIN-18/Ryk represses only VANG-1. These data expand our knowledge of a sophisticated signaling network to control P7.p polarity, and suggests that CHW-1 can alter ligand gradients or receptor priorities in the system.

## Introduction

Rho family small GTPases are intracellular signaling molecules that regulate cytoskeletal rearrangements and transcription, and which affect diverse cellular processes including cell adhesion, polarity, and migration [1]. Historically, the large majority of studies concerning Rho family GTPases have focused on the canonical members RhoA, Rac1, and Cdc42. However, other family members have been identified by strong sequence conservation, and many of them have unique functions [2]. Two of the less studied Rho family GTPases are the closely related Cdc42 subfamily proteins Wnt-regulated Cdc42 homolog-1 (Wrch-1/RhoU) and Cdc42 homologous protein (Chp/Wrch-2/RhoV). Wrch-1 was first identified as a gene whose expression increased when the Wnt signaling pathway was activated [3]. Wrch-1 is thought to be a key Wnt target in oncogenesis, as over-expression of Wnt-1 increases Wrch-1 expression and expression of activated Wrch-1 caused transformation similar to Wnt transformation [3, 4]. Wnt-dependent expression of Wrch-1 does not require β- catenin, but does require c-Jun N-terminal kinase (JNK), implicating the non-canonical Wnt/Planar Cell Polarity (PCP) pathway [3, 5].

The Wnt/PCP pathway guides developing epithelial tissues in orienting their cell divisions in the plane of the epithelium, and thus Wnt/PCP is critical for normal animal development [6, 7]. Misregulation of the Wnt/PCP pathway is linked to cancer development and progression [8, 9]. While the Wnt/PCP pathway is known to utilize Rho family GTPases, including Cdc42, as effectors [10–13], the role of Wrch-1 is unknown. However, a possible role of Wrch-1 in this pathway is consistent with its previously described role in epithelial apical-basal polarity, where Wrch-1 is asymmetrically distributed and binds as an effector the antero-posterior (A-P) and Planar polarity regulating protein Par6 [14, 15].

**Fig 1:**
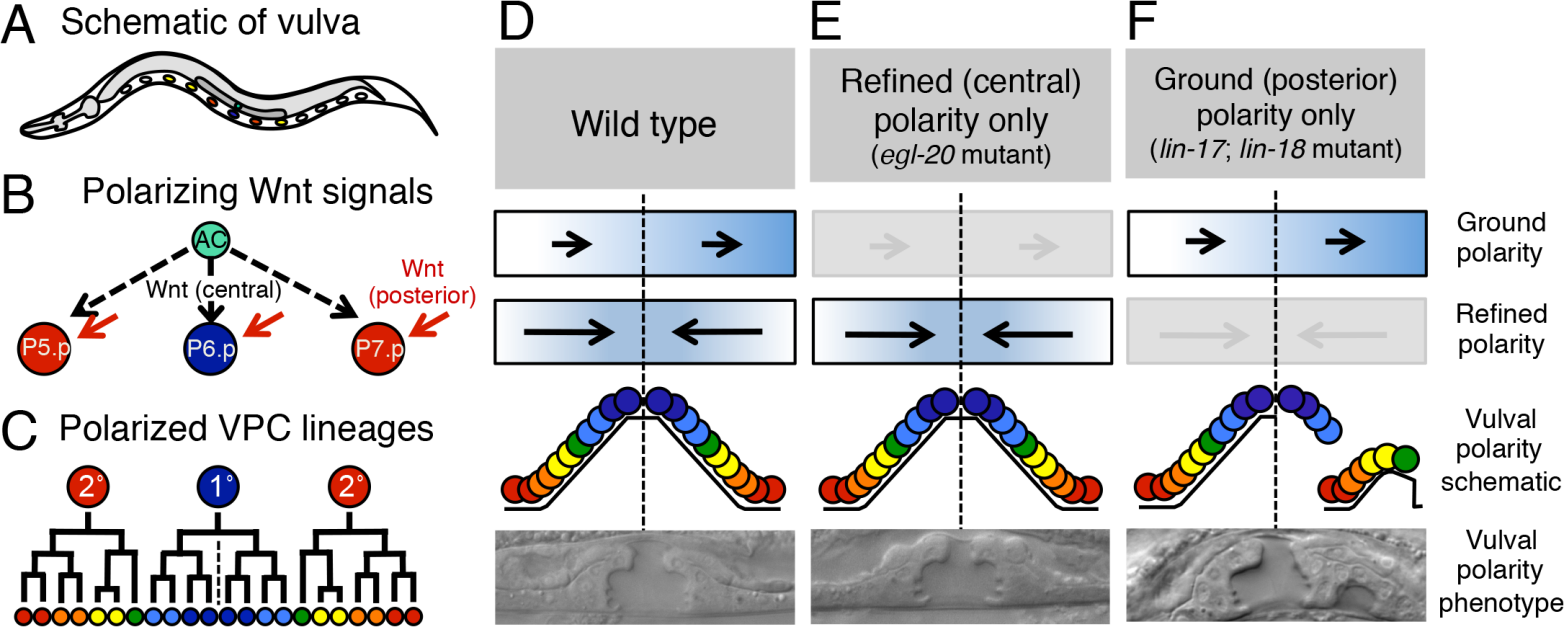
Two Wnt pathways regulate VPC polarization. **A**. Schematic of vulval precursor cells in the whole animal. **B.** Schematic of refined (central) and ground (posterior) Wnt signals regulating VPC polarity. **C.** A schematic of polarized 2°-1°-2° VPC lineages (P5.p (left), P6.p (center), and P7.p (right)). P5.p and p7.p lineages are asymmetric and mirror one another around a central axis (dotted line). (**D-F**) Illustration and images of vulval lineages, model polarity signals and morphology in animals that are (**D**) wild type, (**E**) *egl-20(n585)* using only refined polarity, or (**F**) *lin-17(n671)*; *lin-18(e620)* using only ground polarity. Arrows represent putative Wnt polarity signals received by VPCs, grayed arrows are inactivated polarity signals, and dotted lines represent central vulval axis.

The Wnt/PCP signaling pathway in *C*. *elegans* is implicated largely in anterior- posterior axon guidance and neuronal polarity [16]. Wnt/PCP components VANG-1/Van Gogh, PRKL-1/prickle, FMI-1/flamingo and DSH-1/dishevelled have been shown to function in neuronal polarity and neurite guidance [17, 18], but have also been implicated in other polarity-based events [19, 20]. Additionally, the polarization of cells during development of the vulval structure may involve an analogous mechanism [21]. The vulva develops from the invariant divisions of three vulval precursor cells (VPCs), P5.p, P6.p, and P7.p (Fig 1A; reviewed in [22]). The combination of Wnt, EGF, and Notch signaling pathways instruct P6.p to adopt the 1° cell fate and P5.p and P7.p to adopt the 2° cell fate. The presumptive 1° cell (P6.p) undergoes three cell divisions to generate an A-P symmetrical lineal group of eight cells that forms the central third of the vulva. In contrast, the flanking P5.p and P7.p undergo three divisions to form A-P- asymmetrical lineal groups of seven cells each that form the anterior and posterior thirds of the vulva, respectively. Importantly, P5.p and P7.p must be polarized in opposite directions, with mirror symmetry centered on the non-polarized central P6.p, to generate a functional vulva.

Part of the redundant molecular mechanism of VPC polarization was revealed by loss-of-function mutations in genes encoding the Frizzled receptor (*lin-17*) and the Ryk/Derailed receptor (*lin-18*), which reverse the polarity of P7.p, but not P5.p [23–27].

Redundant actions of diverse Wnt ligands (LIN-44, MOM-2 and CWN-2) also contribute to this process [28]. The phenotype was named P-Rvl (posterior-reversed vulval lineage). Interestingly, loss of two other Wnt homologs, EGL-20 and CWN-1, and orthologs of the PCP components Ror (CAM-1) and Van Gogh (VANG-1) suppress P- Rvl defects caused by removing LIN-17/Fz. Sophisticated control of multiple Wnt ligand gradients has a strong impact on P7.p polarization as well as contribution to vulval induction and competence [29, 30]. In the absence of all Wnt activity, all P7.p cells exhibit the P-Rvl phenotype, revealing that removal of a redundant Wnt signal that confers vulval-oriented polarity reveals an underlying signal that confers posterior- oriented polarity. Further analyses suggest that the posterior-oriented polarity system is governed by mechanistically dissimilar Wnt signaling. [21].

These data suggest a model in which distinct Wnt signals control P5.p and P7.p polarization (Fig 1B) [21]. The first pathway, termed the “ground polarity” signal, polarizes both P5.p and P7.p towards the posterior, and utilizes Wnt/EGL-20, Wnt/CWN-1, Ror/CAM-1, and Van Gogh/VANG-1. A second pathway, termed the “refined polarity” signal, polarizes both P5.p and P7.p towards the centrally located P6.p along a (P-Rvl)-distal (P-D) axis, and utilizes a Wnt signaling pathway involving ligands Wnt/LIN-44 and Wnt/MOM-2 and receptors Fz/LIN-17 and Ryk/LIN-18 [25, 28]. For the sake of clarity, in this study we will refer to “ground (posterior) polarity” and “refined (central) polarity.” Thus, in the normally posteriorly oriented P5.p, ground (posterior) polarity and refined (central) polarity Wnt signals collaborate redundantly to promote the same polarity outcome, and A-P and P-D axes are aligned with the invaginated portion of the 2° lineage oriented towards the 1° lineage, posteriorly. Consequently when single components of either ground (posterior) polarity or refined (central) polarity are lost there are no P5.p A-Rvl phenotypes, because each polarity system maintains P5.p posterior orientation in the absence of the other. In contrast, in P7.p the ground (posterior) polarity and refined (central) polarity Wnt pathways act in opposition; the refined (central) polarity pathway specifying P-D polarity completely overrides the ground (posterior) polarity pathway with high fidelity to orient P7.p anteriorly, towards the 1° lineage and the center of the developing vulva (Fig 1C). Consequently, loss of refined polarity components results in P7.p polarity reversal; loss of either LIN-17/Fz or LIN-18/Ryk displays a partially penetrant P-Rvl phenotype, while loss of both results in 100% P-Rvl [25], suggesting that it is collaboration of LIN-17/Fz and LIN-18/Ryk that interprets external Wnt signals and correctly orients P7.p [21].

Here, we characterize the role of the gene *chw-1*, which encodes the sole *C*. *elegans* ortholog of Chp and Wrch-1. Using an *in vivo* cell migration assay, we present evidence that wild-type CHW-1 signaling is partially constitutively active, unlike most Rho family members, whose activities are tightly regulated. Therefore, transcriptional regulation of CHW-1 may suffice to activate CHW-1-dependent pathways. Using genetic epistasis analysis we show that CHW-1 affects LIN-17/Fz and LIN-18/Ryk differently; loss of *chw-1* suppresses the *lin-17* P-Rvl phenotype, but enhances the *lin-18* P-Rvl phenotype, a pattern not yet described for any other proteins functioning in this system. The contributions of LIN-17/Fz and LIN-18/Ryk to P7.p polarity are unequal, with LIN-17/Fz responsible for roughly two-thirds of the polarizing activity and LIN-18/Ryk for one-third. But in the absence of CHW-1, LIN-17/Fz and LIN-18/Ryk contributions are presumed to be equal. The ground (posterior) polarity receptors CAM-1/Ror and VANG-1/Van Gogh also have genetically distinguishable pathway interactions: loss of CAM-1 suppresses *lin-17* but not *lin-18* mutations, while VANG-1 loss suppresses both *lin-17* and *lin-18* P-Rvl defects. A *chw-1* promoter::GFP fusion construct is excluded from the three VPCs assuming vulval fates, including the polarized P5.p and P7.p, but is expressed in uninduced VPCs. We hypothesize that CHW-1 non-autonomously promotes the contribution of LIN-17/Fz to P7.p polarity at the expense of LIN-18/Ryk contribution.

## Results

### CHW-1 is the *C*. *elegans* ortholog of human Chp/Wrch

**Fig 2:**
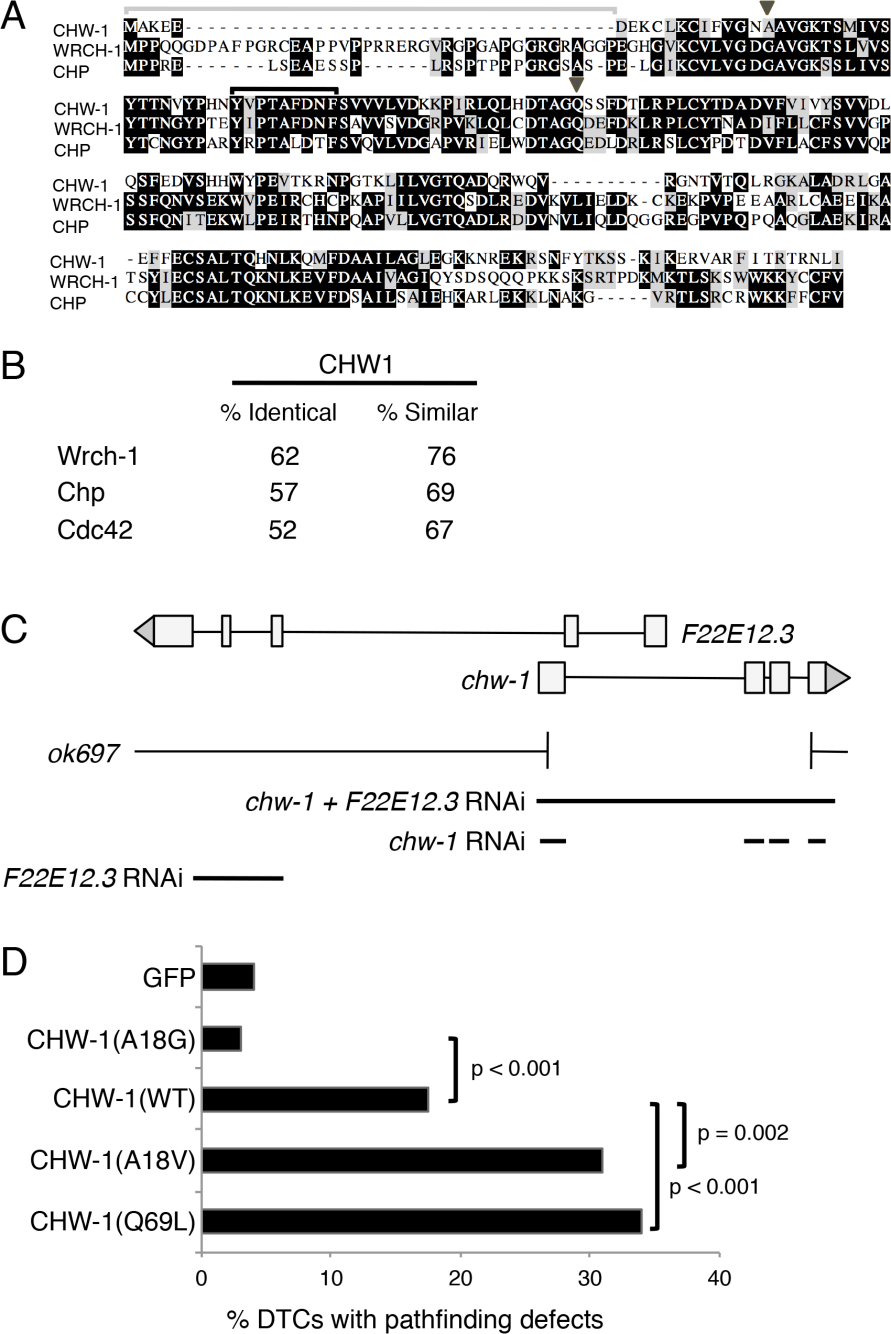
CHW-1 is the *C*. *elegans* ortholog of human Wrch-1/RhoU and Chp/Wrch-2/RhoV. A. Sequence alignment of CHW-1, human Wrch-1, and human Chp. Identical residues have a black background, conservative changes have a gray background, and non-conservative changes have a white background. CHW-1 lacks the N-terminal extension found in Wrch-1 and Chp (gray bracket). CHW-1 also contains an atypical residue (alanine; arrowhead) at position 18 (analogous to position 12 in Cdc42 and most Rho and Ras family members). The core effector domain (black bracket) is most similar to that of Wrch-1, with one conservative variant (V→I) between CHW-1 and Wrch-1. Indicated with a gray caret are the atypical A residue at position 18 and the typical Q residue at position 69, both of which we mutated for our DTC locomotion studies in panel D. **B.** An identity and similarity comparison of the GTPase-domain sequences of CHW-1 with Wrch-1, Chp and Cdc42. **C**. Gene structure of *chw-1* and an overlapping gene prediction (F22E12.3). Below, sequences deleted in *chw-1(ok697)* and sequences used for feeding RNAi are indicated. **D**. An assessment of CHW-1 activity by ectopic expression in DTCs. The *lag-2* promoter was used to drive ectopic CHW-1 expression in DTCs and migration defects were analyzed by DIC microscopy. A total of 200 DTC migrations were analyzed for each construct. Expression of wild-type CHW-1 caused significantly more frequent migration defects than did expression of GFP or CHW-1(A18G). Expression of CHW-1(A18V) or CHW-1(Q61L) caused significantly more DTC migration defects than did expression of WT CHW-1. Tests of statistical significance were performed using Fisher’s exact test.

The *C*. *elegans* genome contains a single gene, F22E12.2, that encodes a protein similar to human Rho family small GTPases Chp/RhoV and Wrch-1/RhoU (Fig 2A–2C). We have named this gene *chw-1* (Chp and Wrch). By amino acid identity, the GTPase domain of CHW-1 is most similar to that of Wrch-1 (Fig 2B). Furthermore, the core effector-binding motifs of CHW-1 and Wrch-1 are nearly identical, while that of Chp has notable differences (Fig 2A). These differences may be reflected by CHW-1 effector selectivity resembling that of Wrch-1 as opposed to Chp. While the CHW-1 GTPase domain is highly conserved, the N- and C-termini are divergent (Fig 2A). First, the N-terminal extension found in Wrch-1 and Chp, which functions to auto-inhibit the proteins (Fig 2A)[31, 32], is mostly absent in CHW-1. Second, whereas Rho family GTPase C-termini generally terminate in CAAX signals for prenyl lipid modifications essential for membrane targeting and protein function [33, 34], Wrch-1 and Chp lack any CAAX motif but terminate instead in CXX motifs that specify palmitoylation and support their membrane targeting and biological activity [4, 31]. However, the CHW-1 protein lacks CAAX, CXX or other lipid modification motifs, suggesting that CHW-1 does not require membrane targeting for its function.

### Native CHW-1 protein may be partially constitutive active

Given these differences, we first assayed CHW-1 activity by testing whether ectopic CHW-1 expression imposed any biological defects. Migration of gonadal distal tip cells (DTCs) is regulated by Rho family GTPases [35, 36], and we have shown that ectopic mutationally activated CED- 10/Rac or CDC-42/Cdc42 were sufficient to perturb migration [37], while control expression of wild-type CED-10/Rac and CDC-42 were phenotypically inconsequential. For these assays we used the *lag-2* promoter [38] to drive expression of CHW-1 or GFP in DTCs and evaluated disrupted DTC migration as a readout of CHW-1 activity. We found that expression of wild-type CHW-1 significantly increased the frequency of errant DTC migration, as compared to expression of GFP (Fig 2D). Wild-type CHW-1 proteins from *C*. *elegans*, *C*. *briggsae* and *C*. *remanei* contain an alanine at position 18, analogous to position 12 in most Ras and Rho small GTPase superfamily members (Fig 2A; S1 Fig). In Wrch-1 and most Rho family GTPases, the wild type residue at this position is a glycine. Mutation of this glycine in small GTPases generally disrupts intrinsic GTPase activity/GAP interaction, thus locking the protein in the GTP-bound state, derepressing the protein and activating downstream signaling. Expression of CHW-1(A18G) abolished the ability of ectopic CHW-1 to cause DTC migration defects, suggesting that the wild-type CHW-1 protein is at least partially active. In contrast, introduction of theoretically stronger activating mutations in residues required for GTPase function [39], A18V and Q69L, disrupted DTC migration more than the wild- type protein (Fig 2D).

Observed gonadal tube-shaped morphology was normal, but the DTC migration was aberrant, as we previously described for defects in CED-10/Rac, and PAK-1 or MAX-2 Pak mutant combinations or ectopically expressed mutationally activated CED- 10/Rac and CDC-42 [37]. In conjunction with this prior study, we hypothesize thatectopically expressing mutationally activated CED-10/Rac, CDC-42 or CHW-1 disrupts spatial recognition of guidance cues, but expression of a non-activated GTPase is neutral, due to the tight regulation of the GTP-GDP cycle typical of Rho family small GTPases [39]. Deletion of *chw-1* (see below) caused no DTC migration defects, nor did we observe P*_chw-1_*::*gfp* expression in the DTCs or other parts of the gonad. Though not an *in vitro* assay of GTP loading of CHW-1, this assay has the benefits of being an *in vivo* assessment of intrinsic CHW-1 activity, particularly since the experiment is controlled with expression of wild-type and A18G mutant proteins, the latter of which compares with similar ectopic expression of wild-type CED-10/Rac and CDC-42 [37]. We hypothesize that the presence of alanine 18 in wild-type CHW-1 confers partially compromised GTP hydrolysis, and hence CHW-1 may be partially activated in the absence of stimulation by GEFs. Thus, expression of CHW-1 may be sufficient to activate CHW-1 effectors. But the ability to mutate the protein to stronger activation argues that CHW-1 retains some GTP hydrolysis capability and negative regulatory input, presumably from a GTPase activating protein (GAP).

### Loss of CHW-1 suppresses the *lin-17* P-Rvl mutant phenotype

Wnt/PCP signaling induces expression of Wrch-1 in vertebrates [5]. Therefore, we hypothesized that CHW-1 expression is likely to function in the Wnt-dependent polarization of vulval precursor cells that bears some resemblance to PCP. However, using the *ok697* deletion mutation that removes most of the *chw-1* coding sequences or RNA interference (RNAi) targeting the *chw-1* genomic region, we detected no vulval polarity defects (Table 1). In double mutant strains, *chw-1(ok967)* suppressed by one-third the P-Rvl defect conferred by two different *lin-17* alleles (Table 1; S2 Fig). The suppression of the *lin-17(*0) P-Rvl phenotype by *chw-1(ok697)* was similar in strength to the suppression of *lin-17(*0) by *vang-1(*0) (VanGogh) and *cam-1(*0) (Ryk), but weaker than suppression of *lin-17(*0) by *egl-20(n585)* (Wnt) [21]. *chw-1(RNAi)* corroborated the *chw-1(ok697)* suppression of the *lin-17(n671)* P-Rvl defect, while control *gfp-*directed RNAi was neutral.

**Table 1:** Loss of CHW-1 suppresses P7.p polarity defects caused by loss of LIN-17/Fz. Animals were grown at 23°C and scored by DIC at late L4 stage. *n* is number of animals scored. Analyses of statistical significance were performed using Fisher’s exact test.

Genotype	% P-Rvl	N	P value
Wild type (N2)	0	78	–
*chw-1(ok697)*	0	142	–
*lin-17(n671)*	72	98	< 0.001^a^
*lin-17(sy277)*	76	187	< 0.001^a^
*lin-17(n671); chw-1(ok697)*	50	117	0.001^b^
*lin-17(sy277); chw-1(ok697)*	48	97	< 0.001^c^
*gfp(RNAi)*	0	44	–
*chw-1(RNAi)*	0	64	–
*lin-17(n671); gfp(RNAi)*	75	52	–
*lin-17(n671); chw-1(RNAi)*	54	89	0.019^d^

^a^ compared to N2

^b^ compared to *lin-17(n671)*

^c^ compared to *lin-17(sy277)*

^d^ compared to *lin-17(n671); gfp(RNAi)*

### Loss of CHW-1 enhances the *lin-18* P-Rvl mutant phenotype

Our observed *lin-17* genetic interactions with *chw-1* were consistent with CHW-1 functioning with EGL-20/Wnt, VANG-1/VanGogh, and CAM-1/Ror to promote refined (central) polarity in opposition to ground (posterior) polarity. Previous studies suggested that the partially redundant refined (central) polarity receptors LIN-17/Fz and LIN-18/Ryk function in pre- empting EGL-20/Wnt-mediated ground (posterior) polarity [21, 25]. Therefore we tested the hypothesis that CHW-1 functions in ground (posterior) polarity by examining the genetic interaction between *chw-1* and *lin-18* mutations. Approximately 35% of *lin-18(e620)* animals are P-Rvl [25], and we corroborated this observation with *lin-18(n1051)* (Table 2; S3 Fig). Counter-intuitively, we found that loss of *chw-1* enhanced rather than suppressed P-Rvl defects conferred by loss of *lin-18* (Table 2), suggesting that CHW-1 does not function as part of the ground (posterior) polarity program in opposition to refined (central) polarity.

**Table 2:** Loss of CHW-1 enhances P7.p polarity defects caused by loss of LIN-18/Ryk. Animals were grown at 23°C and scored by DIC at late L4 stage. *n* is number of animals scored. Analyses of statistical significance were performed using Fisher’s exact test.

Genotype	% P-Rvl	N	P value
Wild type (N2)	0	78	–
*chw-1(ok697)*	0	142	–
*lin-18(e620)*	34	174	< 0.001^a^
*lin-18(n1051)*	35	236	< 0.001^a^
*chw-1(ok697); lin-18(e620)*	50	94	0.013^b^
*chw-1(ok697); lin-18(n1051)*	52	131	0.002^c^
*gfp(RNAi)*	0	44	–
*chw-1(RNAi)*	0	64	–
*gfp(RNAi); lin-18(e620)*	33	67	–
*chw-1(RNAi); lin-18(e620)*	47	101	0.041^d^

^a^ compared to N2

^b^ compared to *lin-18(e620)*

^c^ compared to *lin-18(n1051)*

^d^ compared to *lin-18(e620); gfp(RNAi)*

Interpretation of *chw-1* genetic interactions with *lin-17* and *lin-18* was complicated by the structure of the overlapping gene, F22E12.3, whose first two exons are predicted to lie within intron 1 of *chw-1*. In addition to disrupting *chw-1*, the *ok697* deletion removes these two F22E12.3 exons (wormbase.org release WS246; Fig 2C). Similarly, the original library clone for bacterially mediated feeding RNAi (“*chw-1* + F22E12.3 RNAi”)[40] targets both *chw-1* and the first two exons of F22E2.3. Therefore, it is theoretically possible that the observed suppression of *lin-17* P-Rvl and enhancement of *lin-18* P-Rvl phenotypes was due to loss of *chw-1*, F22E12.3, or both. We resolved this issue by using F22E12.3-specific feeding RNAi, which failed to suppress *lin-17* or enhance *lin-18* mutants (Table 3). Conversely, we constructed *chw-1*-directed feeding RNAi targeting only the full-length *chw-1* cDNA, and this RNAi suppressed *lin-17* and enhanced *lin-18* mutants. We conclude that it is loss of *chw-1* that caused *lin-17* suppression and *lin-18* enhancement.

**Table 3:** Effects on P7.p polarity are *chw-1*-specific. Animals were grown at 23°C and scored by DIC at late L4 stage. *n* is number of animals scored. Data for strains marked with an asterisk (*) are from Table 1 (*lin-17*) or Table 2 (*lin-18*). Analyses of statistical significance were performed using Fisher’s exact test.

Genotype	% P-Rvl	N	P value
lin-17(n671)*	72	98	–
*lin-17(n671)*; chw-1(ok697)*	50	117	0.001^a^
lin-17(n671); gfp(RNAi)*	75	52	0.847^a^
*lin-17(n671)*; chw-1(RNAi)*	54	89	0.019^b^
*lin-17(n671); F22E12*.*3(RNAi)*	72	54	0.827^b^
*lin-17(sy277)*; *chw-1+F22E12*.*3(RNAi)*	51	78	0.010^b^
lin-18(e620)*	34	174	–
*chw-1(ok697)*; lin-18(e620)*	50	94	0.013^c^
*gfp(RNAi)*; lin-18(e620)*	33	67	0.880^c^
*chw-1(RNAi)*; lin-18(e620)*	47	101	0.041^d^
*F22E12*.*3(RNAi)*; lin-18(e620)*	3`	42	0.856^d^
*chw-1+F22E12*.*3(RNAi)*; lin-18(e620)*	48	80	0.051^d^

^a^ compared to *lin-17(n671)*

^b^ compared to *lin-17(n671); gfp(RNAi)*

^c^ compared to *lin-18(e620)*

^d^ compared to *lin-18(e620); gfp(RNAi)*

Taken together, our results 1) corroborate that LIN-17/Fz and LIN-18/Ryk are both quantitatively non-equivalent in directing refined (central) vulval polarity and 2) suggest that the function of CHW-1 is to equalize the signaling contributions of these two refined (central) polarity Wnt receptor signals.

As reported, partially redundant LIN-17/Fz and LIN-18/Ryk functions govern refined (central) polarity, such that loss of both LIN-17/Fz and LIN-18/Ryk results in a 100% P-Rvl defect, as all P7.p cells orient toward the sole remaining Wnt signal, the Egl-20/Wnt-mediated ground (posterior) polarity pathway. Consequently, loss of EGL- 20/Wnt in the *lin-17*; *lin-18* double mutant background resulted in suppression (and randomization) of the P-Rvl phenotype to ~50%, and occurrence of low numbers of A- Rvl and A+P-Rvl animals. Similarly, loss of CWN-1/Wnt, a minority contributor to the EGL-20/Wnt ground (posterior) polarity pathway, in a *lin-17*; *lin-18* double mutant results in suppression of the P-Rvl phenotype weaker than that observed for loss of EGL- 20/Wnt. Thus, loss of ground (posterior) polarity components suppressed the 100% P- Rvl phenotype of the *lin-17*; *lin-18* double mutant [21]. To further test whether CHW-1 functions as part of the EGL-20/Wnt (and CWN-1/Wnt) ground (posterior) polarity pathway, we constructed the *lin-17*; *chw-1*; *lin-18* triple mutant strain. We found no suppression of the 100% P-Rvl phenotype by *chw-1(ok697)* (Table 4), suggesting that CHW-1 does not play a substantial role in ground (posterior) polarity signaling.

**Table 4:** Loss of CHW-1 does not disrupt ground polarity. Animals were maintained at 23°C and scored by DIC at late L4 stage. *n* is number of animals scored. Analyses of statistical significance were performed using Fisher’s exact test.

Genotype	% P-Rvl	n	P value
*lin-17(n671); lin-18(e620)*	100	67	–
*lin-17(n671); chw-1(ok697); lin-18(e620)*	100	78	–
*egl-20(n585)*	0	52	–
*egl-20(n585); chw-1(ok697)*	0	198	–
*lin-17(n671)*	72	98	–
*lin-18(e620)*	34	174	–
*lin-17(n671); egl-20(n585)*	11	157	< 0.001^a^
*egl-20(n585); lin-18(e620)*	6	67	< 0.001^b^
*lin-17(n671); egl-20(n585); chw-1(ok697)*	8	86	0.654^c^
*egl-20(n585); chw-1(ok697); lin-18(e620)*	0	75	0.047^d^

^a^ compared to *lin-17(n671)*

^b^ compared to *lin-18(e620)*

^c^ compared to *lin-17(n671); egl-20(n585)*

^d^ compared to *egl-20(n585); lin-18(e620)*

To test further whether CHW-1 functions in parallel to EGL-20/Wnt, we built *chw- 1* triple mutants with *lin-17*; *egl-20* or *egl-20*; *lin-18*. Loss of CHW-1 did not cause a synthetic phenotype in a double mutant combination with *egl-20(n585)*, and did not suppress the *lin-17*; *egl-20* double mutant (Table 4), suggesting that loss of Egl-20/Wnt blocks the ability of *chw-1(*0) suppression. In contrast, loss of CHW-1 weakly suppressed the *egl-20*; *lin-18* double mutant phenotype, arguing that CHW-1 can regulate LIN-17/Fz activity in the absence of EGL-20/Wnt. Notably, although loss of CHW-1 enhanced the *lin-18* P-Rvl single mutant defect, loss of CHW-1 did not enhance the *egl-20*; *lin-18* double mutant P-Rvl defect. Therefore, though CHW-1 does not appear to be involved in the ground (posterior) polarity signal, EGL-20/Wnt activity may be required for *chw-1(ok697)* enhancement of LIN-18/Ryk loss.

### CHW-1 function may be non-autonomous

**Fig 3:**
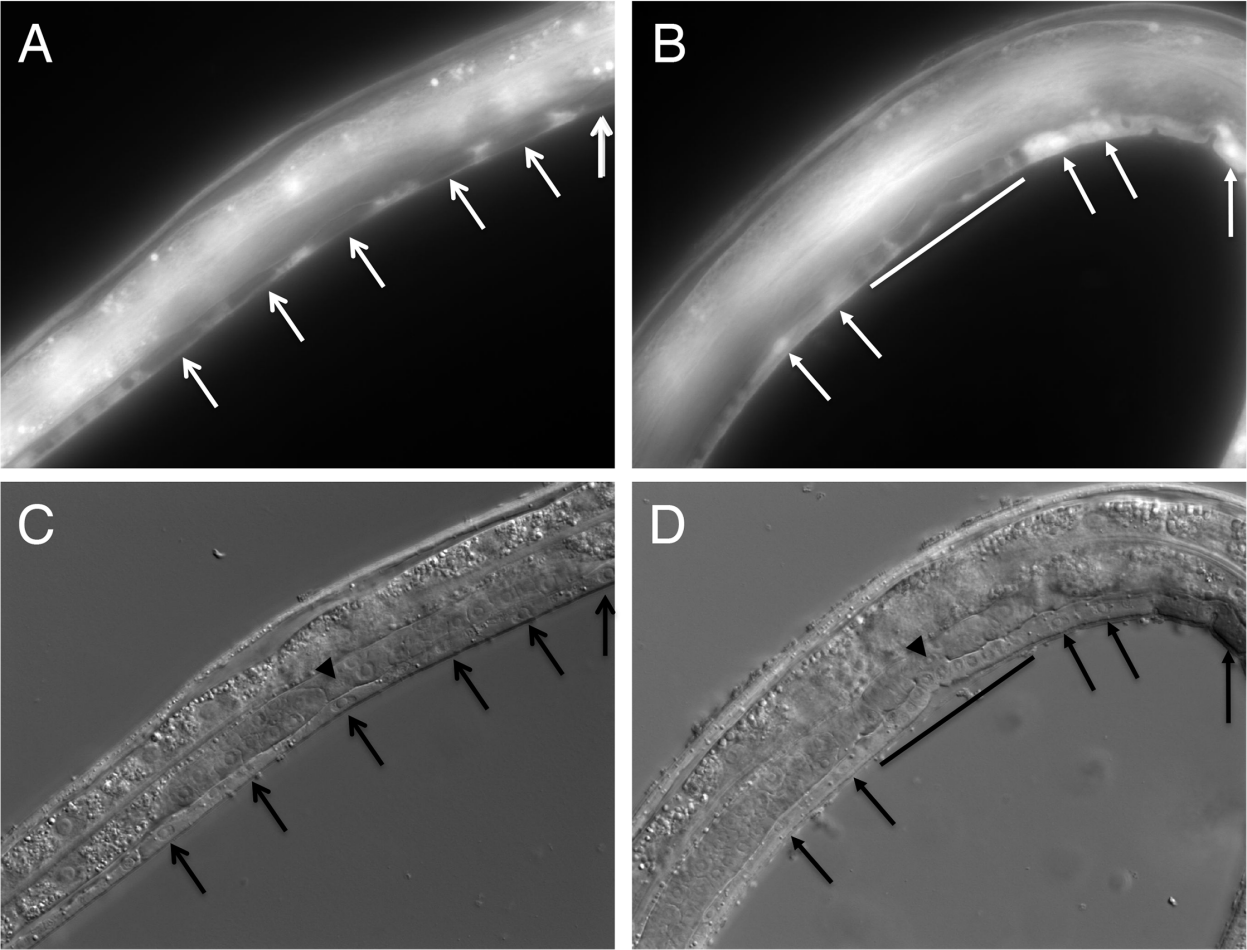
*chw-*1 promoter-driven GFP is absent from induced VPCs during vulval development, but is expressed in uninduced, 3° cells after induction. **A, B.** Fluorescent images (600x) of a *reIs3[*P*_chw-1_*::*gfp]*-bearing animals. **C, D.** DIC images corresponding to A and B, respectively. A and C show an animal at the Pn.p (undivided) stage of vulval development prior to. VPCs (Pn.p cells) are indicated by white arrows in A and black arrows in C, and the anchor cell is indicated by a black arrowhead. B and D show an animal at the Pn.pxx (2 divisions) stage during ingression onset of induced VPC progeny, just prior to the last round of division. Differentiated vulval progeny starting to coalesce and ingress are indicated by a white bar in B and a black bar in D. Uninduced 3° cells have divided once and are indicated by solid white arrows in B and solid black arrows in D. Note that 3° cells express GFP, with stronger nuclear GFP signal due to a single nuclear localization signal in the construct. Induced vulval lineages (1°s and 2°s) do not express GFP. In both animals anterior is right, ventral is down.

To assess the endogenous CHW-1 expression pattern, we constructed a plasmid with *chw-1* promoter sequences driving green fluorescent protein (GFP), and generated the *reIs3* transgene containing this plasmid. Prior to vulval induction, the *reIs3* P*_chw-1_*::*gfp*-containing transgene failed to express GFP in the six VPCs, but is visible in surrounding cells (Fig 3A and 3B). Upon EGF induction GFP was expressed in non-vulval (3°) VPC lineages and surrounding cells, but was excluded from VPC lineages that had been induced to form vulva (P5-7.p and subsequent 2°-1°-2° lineages; Fig 3C and 3D). The exclusion extended throughout larval development, and all expression faded after the L4 stage.

We hypothesized that Wnt pathway activity might actively exclude GFP and presumably CHW-1 expression from vulval lineages, and tested this hypothesis with single and double mutant combinations harboring the *reIs3*[P*_chw-1_*::*gfp*] transgene. However, all genotypes tested failed to alter GFP expression to vulval lineages. We tested *lin-17(n671)*, *lin-18(e620)*, *cam-1(gm122)*, and *vang-1(ok1142)* single mutant animals harboring the transgene, as well as *cwn-1(ok546)*; *egl-20(n585)* double mutant (Wnts required for ground (posterior) polarity) and *lin-17(n671)*; *lin-18(e620)* double mutant combinations. We also tested Wnt signaling mutants *bar-1(ga80)* (encoding β- catenin) and *pry-1(mu38)* (encoding axin). No mutant combination had an effect on GFP expression (S3 Fig).

It is difficult to reconcile P*_chw-1_*::*gfp* expression with LIN-17/Fz and LIN-18/Ryk activity in VPCs. Perhaps CHW-1 non-autonomously regulates Wnt ligand activity, and thus loss of CHW-1 has differential effects on *lin-17* and *lin-18* mutants. Alternatively, our promoter fusion construct may not accurately represent endogenous CHW-1 expression. We were limited in our inclusion of upstream promoter sequences by transgene toxicity; we were unable to isolate live transgenic animals harboring constructs containing F22E12.3 exon 3 or further upstream sequences, or *chw-1* intron 1, implying the existence of regulatory elements that titrate essential transcription factors. This genomic region also harbors non-coding RNA genes F22E12.7 and F22E12.13, whose over-expression could also cause toxicity.

It has been reported that T-cell acute lymphoblastic leukaemia, a strongly Notch- dependent cancer, up-regulates Wrch-1/RhoU in a Notch activity-dependent manner to promote cell migration and chemotaxis [41]. A large set of putative *C*. *elegans* Notch- LAG-1/CSL transcriptional client genes were identified computationally [42]. We searched this dataset, but the *chw-1* promoter was not identified as a putative Notch transcriptional target, consistent with our *reIs3* reporter not evincing expression in 2° cells.

### LIN-17/Fz antagonizes both VANG-1/VanGogh and CAM-1/Ryk, while LIN-18/Ryk antagonizes only VANG-1/VanGogh

**Fig 4:**
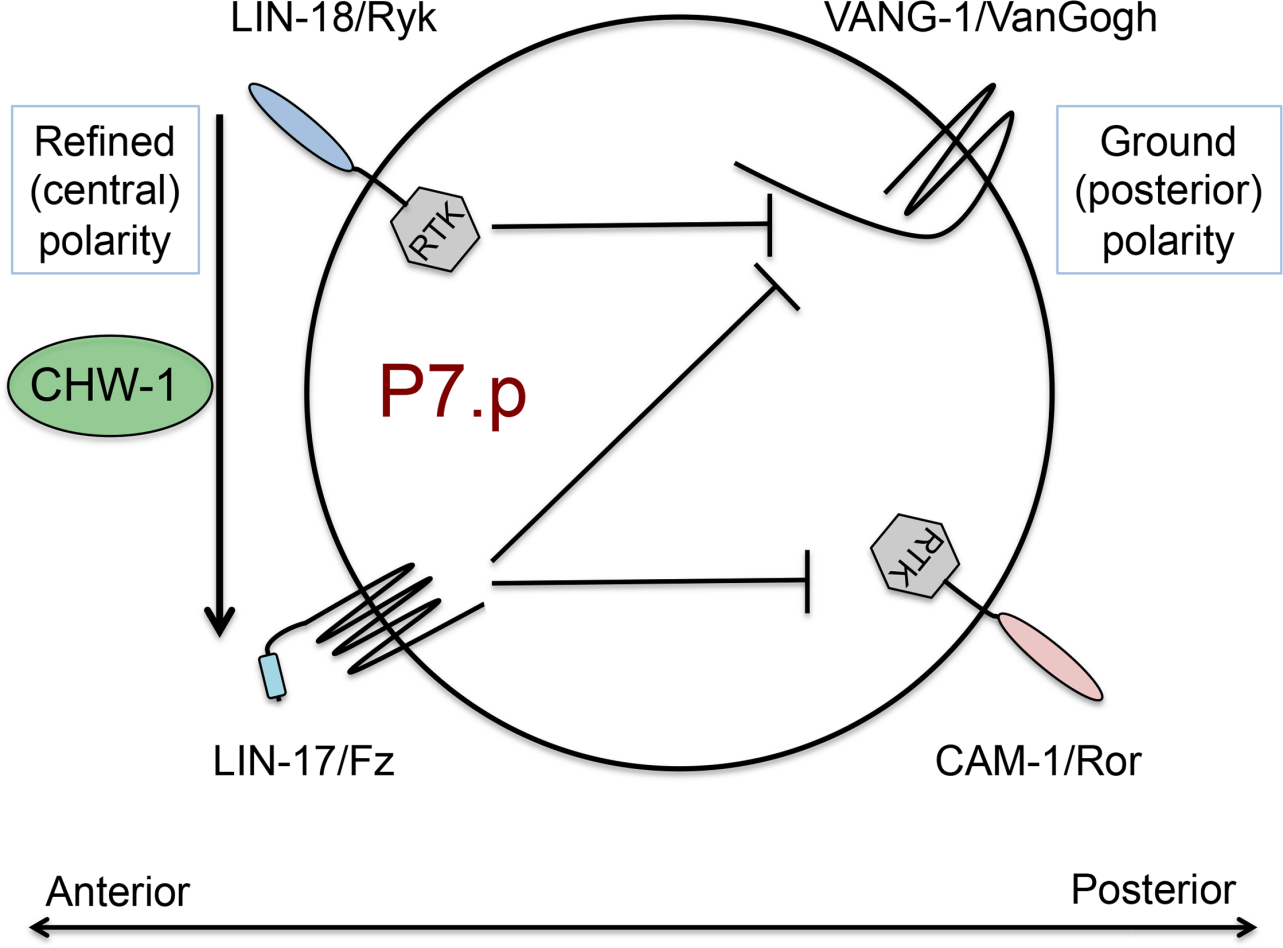
CHW-1 regulates relative LIN-17/Fz and LIN-18/Ryk signaling in refined (central) polarity, and LIN-17/Fz and LIN-18/Ryk execute a specific repressive program to exclude activity of the ground (posterior) polarity system. Shown is a schematic of P7.p with anterior, the direction of refined (central) polarization, to the left and posterior, the direction of ground (posterior) polarization to the right.

Consistent with their acting as receptors for EGL-20/Wnt and CWN-1/Wnt in the ground (posterior) polarity signal, loss of CAM-1/Ror or VANG-1/VanGogh suppressed the *lin-17* (Fz) mutant P-Rvl mutant phenotype [21] (Table 5 lines 3 vs. 2, 8 vs. 7 and 12 vs. 11, respectively; S4 Fig), but *cam-1* and *vang-1* mutant suppression of *lin-18* mutations was not tested. We found that disruption of *vang-1* suppressed the *lin-18(e620)* P-Rvl phenotype (Table 5, lines 21 vs. 20), but *cam-1(gm122)* failed to suppress either *lin-18* allele (Table 5, lines 16, 17 vs. 15). Collectively, the LIN-17/Fz and LIN-18/Ryk refined (central) polarity signals need to overwhelm the VANG-1/VanGogh and CAM-1/Ror ground (posterior) polarity signals to correctly polarize P7.p. We therefore hypothesize that LIN-18 signal represses VANG-1/VanGogh, while LIN-17/Fz represses both VANG-1/VanGogh and CAM-1/Ror (Fig 4). Thus, in the absence of LIN-18/Ryk VANG-1 signaling is elevated, and so mutation of VANG-1 abolishes that signal. Since CAM-1/Ror is not repressed by LIN-18/Ryk, further CAM-1/Ror mutation in a LIN-18/Ryk mutant background has no effect. Conversely, if LIN-17/Fz represses both VANG-1 and CAM-1/Ror, LIN-17/Fz mutants are partially suppressed by loss of either.

**Table 5:** Loss of VANG-1 but not CAM-1/Ror in the *lin-17* (Fz) mutant background retains sensitivity to CHW-1 activity. Also, loss of CAM-1 but not VANG-1 in the *lin-18* (Ryk) background retains sensitivity to CHW-1 activity. In other words, VANG-1 loss abolishes the ability of *lin-18* mutant animals to respond to *chw-1* mutation or RNAi. Animals were maintained at 23°C and scored by DIC at late L4 stage. *n* is number of animals scored. Analyses of statistical significance were performed using Fisher’s exact test.

	Genotype	% P-Rvl	N	P Value
1	*cam-1(gm122)*	0	77	–
2	*lin-17(n671)* ^§^	72	113	–
3	*lin-17(n671); cam-1(gm122)* ^§^	46	54	0.005
4	*lin-17(n671); cam-1(gm122); gfp(RNAi)*	49	135	–
5	*lin-17(n671); cam-1(gm122); chw-1(RNAi)*	47	116	–
6	*vang-1(ok1142)*	0	46	–
7	*lin-17(n671)*	72	98	–
8	*lin-17(n671); vang-1(ok1142)* ^§^	48	60	0.005
9	*lin-17(n671); gfp(RNAi); vang-1(ok1142)*	49	139	–
10	*lin-17(n671); chw-1(RNAi); vang-1(ok1142)*	34	172	0.008^a^
11	*lin-17(n671); gfp(RNAi)*	68	158	–
12	*lin-17(n671); vang-1(RNAi)*	45	159	<0.001^b^
13	*lin-17(n671); chw-1(ok697); gfp(RNAi)*	50	296	–
14	*lin-17(n671)*; *chw-1(ok697)*; *vang-1(RNAi)*	35	212	<0.001^c^
15	*lin-18(n1051)* ^§^	35	236	–
16	*cam-1(gm122); lin-18(n1051)*	35	77	–
17	*cam-1(gm122); lin-18(e620)*	38	63	–
18	*cam-1(gm122); gfp(RNAi); lin-18(e620)*	30	101	–
19	*cam-1(gm122); chw-1(RNAi); lin-18(e620)*	50	163	<0.001^d^,^e^
20	*lin-18(e620)*	34	174	–
21	*vang-1(ok1142) lin-18(e620)*	3	121	<0.001^f^
22	*vang-1(ok1142) lin-18(e620); gfp(RNAi)*	0	87	–
23	*chw-1(RNAi)*; *vang-1(ok1142) lin-18(e620)*	4	116	–
24	*chw-1(ok697); vang-1(ok1142) lin-18(e620)*	8	71	–

^a^ compared to *lin-17(n671); gfp(RNAi); vang-1(ok1142)*

^b^ compared to *lin-17(n671); gfp(RNAi)*

^c^ compared to *lin-17(n671); chw-1(ok697); gfp(RNAi)*

^d^ compared to *cam-1(gm122); lin-18(e620)*

^e^ compared to *cam-1(gm122); gfp(RNAi); lin-18(e620)*

^f^ compared to *lin-18(e620)*

^§^ Data previously published [21]

We also found that loss of CHW-1 had different effects in *cam-1* or *vang-1* mutant backgrounds (Table 5). *chw-1*-directed RNAi did not alter suppression of *lin-17*; by *cam-1* in the triple mutant compared to the *gfp*-directed RNAi control (Table 5, line 5 vs. 4). In contrast, *chw-1*-directed RNAi was additive with *vang-1* in suppressing the *lin- 17* P-Rvl phenotype compared to the *gfp* RNAi baseline: the *chw-1* or *vang-1* single mutant or RNAi suppressed the *lin-17* mutant, while the *chw-1*; *vang-1* double knockout suppressed the *lin-17* mutant even further (Table 5, lines 8 vs. 9 vs. 10 and 12 vs. 13 vs. 14). We note that the degree of the *lin-17*; *chw-1*; *vang-1* P-Rvl phenotype, 35%, is the same as the *lin-18* single mutant phenotype, also 35%. Whether this value represents a coincidence or reflects a fundamental property of the signaling system is unknown.

We additionally observed that P-Rvl phenotype of *cam-1*; *lin-18* mutant animals we enhanced by *chw-1* loss (Table 5, lines 18 vs. 19). Yet the P-Rvl phenotype of *vang-1 lin-18* was unaltered by *chw-1* loss (Table 5, lines 21 vs. 22 vs. 23 vs. 24). Based on these interactions we can make models interpreting CHW-1 interactions with LIN-17 and LIN-18, and also models interpreting LIN-17 and LIN-1 interactions with VANG-1 and CAM-1, but we cannot reconcile both models with the data we have. We propose that missing information is required to make sense of these inconsistencies. However, we still consider the model that CHW-1 promotes LIN-17/Fz signaling at the expense of LIN-18/Ryk signaling to best describe the data.

## Discussion

In the absence of CHW-1, the relative contributions of LIN-17/Fz and LIN-18/Ryk to P7.p polarity appear to be roughly equivalent. We therefore hypothesize that CHW-1 regulates relative contribution of LIN-18/Ryk and LIN-17/Fz receptors to the refined (central) polarity decision, or to antagonism of the ground (posterior) polarity decision, which may be the same thing. We propose that CHW-1 boosts LIN-17/Fz signaling at the expense of LIN-18/Ryk, or shifts ligand signaling to favor LIN-17/Fz activation over LIN-18/Ryk activation. Thus, in a *lin-17* mutant loss of CHW-1 increases LIN-18/Ryk contribution, thus partially compensating for LIN-17/Fz loss. Conversely, in a *lin-18* mutant, loss of CHW-1 decreases LIN-17/Fz contribution, thus exacerbating the *lin-18* mutant P-Rvl phenotype. It is unknown why P7.p would need non-equivalent signaling of these diverse Wnt receptor systems, or mechanistically how CHW-1 could act to make their contributions nonequivalent.

Use of unexpected signaling input to regulate receptor activity or output has multiple precedents in growth cone guidance. The UNC-40/DCC and SAX-3/Robo transmembrane receptors, which canonically regulate dorsal-ventral axon guidance in response to UNC-5/Netrin and SLT-1/Slit ligands, respectively, can be modulated by VAB-8/kinesin-related, UNC-73/RhoGEF and MIG-2/RhoG signaling input to regulate anterior-posterior migrations instead [43, 44]. The EVA-1 co-receptor alters SAX-3/Robo interactions with UNC-40/DCC signaling [45], and the UNC-129/TGF-β-related protein regulates UNC-6/Netrin outcomes through UNC-40/DCC or UNC-5/Netrin receptors [46]. We hypothesize that CHW-1 could perform a similar function in regulating LIN- 17/Fz versus LIN-18/Ryk receptor contributions to P7.p polarity. CAM-1/Ror and VANG- 1/Van Gogh receptors also contribute differentially to LIN-17/Fz versus LIN-18/Ryk signaling in P7.p polarity, and the interactions of these pathways are likely complex.

However, these mechanisms are cell autonomous, and our data suggest that CHW-1 functions non-autonomously, though combined early non-vulval and later 3°- specific expression preclude the use of heterologous promoter expression of CHW-1 to conclusively resolve this question. If CHW-1 function is non-autonomous, we hypothesize that it controls the composition of Wnt gradients. There is a precedent for control Wnt gradients impacting P-Rvl phenotypes. CAM-1/Ror has been argued to sequester Wnts, thus impacting P-Rvl phenotype [21]. This phenomenon is so pronounced that even anterior displacement of the CAM-1/Ror-expressing canal- associated neurons (CANs) and their axons disrupts Wnt gradients and impacts the P- Rvl phenotype. This observation led to the intriguing model that alteration of nervous system architecture impacts subsequent epithelial cell development [29]. With this in mind we scrutinized *chw-1(ok697)* mutant animals using pan-neural and CAN/HSN GFP markers, but observed no alterations in nervous system structure, CAN axon extension or CAN cell migration. FGF was found to induce the SM cells to secrete CWN-1/Wnt, thus contributing to P7.p polarization [47]. We did not observe GFP expression in the SMs using the *reIs3* transgene. Additional players in this system are the PAK-1 S/T kinase, NCK-1/Nck adaptor, and CED-10/Rac [48]. The PAK S/T kinase is an established effector of mammalian Chp/Wrch [3, 32, 49–51], [52], suggesting that *C*. *elegans* PAK-1 could function as a CHW-1 effector. Thus CHW-1 may signal through a different cascade than the implied Rac-Pak signal or interact somehow with PAK-1.

VANG-1/VanGogh and CAM-1/Ror respond to LIN-44 and MOM-2 Wnts to establish ground (posterior) polarity. Cooperative LIN-17/Fz and LIN-18/Ryk respond to EGL-20 and CWN-1 Wnts to override the ground (posterior) polarity system to orient P7.p anteriorly towards the Anchor Cell, thus comprising the basics of the refined (central) polarity system (Fig 1; 21). The genetic interactions among LIN-17/Fz, LIN- 18/Ryk, VANG-1/VanGogh and CAM-1/Ror transmembrane protein systems can be incorporated into a formal, repressive P7.p polarity model of refined (central) polarity and ground (posterior) polarity (Fig 4). We present inferred LIN-17/Fz and LIN- 18/Ryk repressive activities, but not functions of VANG-1 and CAM-1/Ror, since they cannot be inferred by our genetics results. In Fig 4 VANG-1 and CAM-1 are arbitrarily placed above/below; their placement is not meant to infer specific VANG-1 or CAM-1 outputs. Loss of LIN-18/Ryk was suppressed by mutation of VANG-1 but not CAM-1/Ror (Table 5). Consequently, we infer that VANG-1 is derepressed by LIN- 18/Ryk loss, thus re-directing P7.p posteriorly in the LIN-18/Ryk mutant background.

And so concomitant VANG-1 loss suppressed the LIN-18/Ryk mutant phenotype. Since CAM-1/Ror loss did not suppress the LIN-18/Ryk mutant, we infer that CAM-1 is not repressed by LIN-18/Ryk loss. Conversely, loss of LIN-17/Fz is suppressed by mutation of either CAM-1/Ror or VANG-1, and furthermore the *vang-1*; *cam-1* double mutant additively represses the *lin-17* mutant more than either single mutant. We hypothesize that LIN-17/Fz represses both VANG-1 and CAM-1/Ror; loss of LIN-17/Fz derepressed both VANG-1 and CAM-1/Ror, and hence additive *vang-1* and *cam-1* loss additively represses the *lin-17* P-Rvl phenotype. Since the *lin-17* P-Rvl phenotype is not completely additively suppressed by the *vang-1*; *cam-1* double mutant, we hypothesize the existence of a third ground (posterior) polarity signal, also suppressed by LIN-17/Fz, in parallel to VANG-1 and CAM-1/Ror posterior-orienting signals (not shown).

We also incorporate the CHW-1 modulating activity into this model, proposing that CHW-1 promotes activation of LIN-17/Fz at the expense of LIN-18/Ryk. Given the observed GFP expression pattern driven by transgenic *chw-1* promoter with *reIs3*, we hypothesize that CHW-1 functions non-autonomously to impact multiple Wnt gradients or receptor activity. There exist two precedents for non-autonomous impacts on refined (central) polarity Wnt gradients. FGF secreted by the induced presumptive 1° VPC, P6.p, induced the SMs, neighboring the Anchor Cell, to secrete Wnts in support of AC polarizing signal [47]. Nervous system development and placing of potential Wnt sinks like the CANs and their axons can also sculpt Wnt gradients, thus impacting refined (central) polarity and perhaps ground (posterior) polarity [29]. It is currently unclear how CHW-1 fits into this paradigm, but future studies will fit CHW-1 amongst the many emerging players.

## Materials and Methods

### Nematode strains and analysis

*C*. *elegans* strains were cultured using standard techniques [53]. All strains used were derivatives of wild-type Bristol strain N2 and were maintained at 23°C.

### Genetics and RNAi

*chw-1(ok697)* homozygotes were identified by PCR using primers TK12 (5’-aatctttgtcgccacgtaatca), TK13 (5’-aaccgcagaaaaagcaaaagag), and TK14 (5’-ccggcaatctaaaattgaagga). This combination amplifies a 638 bp product (TK12/TK14) when *chw-1(+)* is present and an 818 bp product (TK12/TK13) when *chw-1(ok697)* is present (see S2 Fig).

When constructing double mutants between *lin-17* and *chw-1* or *egl-20*, *lin-17* was balanced with the GFP-tagged *hT2 qIs48[Pmyo-2*::*GFP+Ppes-10*::*GFP+gut*::*GFP]* (I;III) translocation and homozygotes were identified as GFP negative. When constructing double mutants with *lin-18(e620)*, candidate homozygotes were identified based on the P-Rvl phenotype, and the *lin-18(e620)* molecular lesion was confirmed by HpyCH4V digestion of a PCR product generated using primers TK25 (5’- gcaaacatcgactacctctcg) and TK26 (5’-ccgagcctctcttcaagtttt). Digestion of the *lin-18(+)* PCR product yields bands of 270, 190, and 40 bp, while digestion of the *lin-18(e620)* PCR product yields bands of 460 and 40 bp (S3 Fig). *cam-1(gm122)* homozygotes were selected based on the withered tail (Wit) phenotype [54].

*vang-1(ok1142)* was detected using primers TK29 (5’-tgaccagattttcaaccgaaat), TK30 (5’-aaaagctttgaaccgccataa), and TK32 (5’-gcttgctcggtcaaattgaag). This combination amplifies a 1121 bp product (TK29/TK30) when *vang-1(+)* is present and a 1035 bp product (TK29/TK32) when *vang-1(ok1142)* is present (S4 Fig).

Bacterial-mediated RNAi was performed essentially as described previously [55]. Briefly, L4 animals were placed on a lawn of *E*. *coli* (strain HT115(DE3)) expressing dsRNA from plasmid L4440, and their progeny were scored at the L4 stage. RNAi constructs targeting both *chw-*1 and *F22E12*.*3* (Wormbase RNAi ID WBRNAi000113757) were described previously [40]. Constructs to specifically target either *chw-*1 or *F22E12*.*3* were generated for this work (details below). A construct targeting GFP was used as a negative control [56].

### Phenotypic analysis

Defects in DTC migration were scored at the late L4 stage using DIC microscopy. Each gonad arm was analyzed separately and was considered abnormal if the gonad arm deviated from the typical “U” shape [57]. The significance of differences in the frequency of DTC migration defects was analyzed using Fisher’s exact test.

Vulval phenotypes were scored at the mid-L4 larval stage using DIC microscopy. Animals were classified as P-Rvl if we observed an ectopic posterior invagination that was separated from the primary vulval invagination by adherent cells. Additionally, we examined the putative reversed P7.p lineage for number of cells, to account for the possibility of ectopic induction. None was found. For some genotypes, we did observe a small number of animals (<2%) with abnormal vulval morphology distinct from VPCpolarization. These animals were included in the normal category because VPCs were properly polarized. The significance of differences in P-Rvl frequency between strains was analyzed using Fisher’s exact test.

### Plasmids and transgenes

To generate a *chw-1*-specific RNAi construct, the entire *chw-1* ORF was amplified from oligo-dT primed cDNA and cloned into pBluescript SK+. The *chw-1* ORF was then shuttled into the bacterial-mediated RNAi plasmid, pPD129.36 (L4440; A. Fire, personal communication), using *Not I* and *Sal I*. We attempted to clone a full F22E12.3 ORF, but were unable to amplify any F22E12.3 fragments from cDNA. Therefore, an F22E12.3-specific feeding RNAi construct was generated by cloning a PCR fragment amplified from genomic DNA lacking *chw-1* coding sequences into pPD129.36 using *Bgl II*. The F22E12.3 PCR product was amplified from cosmid DNA (F22E12) using primers TK35 (5’-ttttagatctttgcctttgtaggctggatt) and TK36(5’-ttttagatctttccgacattaattggaaattg). All inserts were confirmed by sequencing.

To express chw-1 cDNA ectopically in DTCs, we used the pJK590 vector, which contains *lag-2* promoter sequences that drive expression in DTCs [38]. To clone *chw-1* into pJK590, we first used site-directed mutagenesis to eliminate a *Bsm I* site in the promoter (pJK590-mut), thus allowing use of the polylinker *Bsm I* site for cloning the *chw-1* cDNA. We then amplified *chw-1* cDNA yk1263f9 with primers DJR543 (AAAAAAaccggtGGCCGGCC**gaaaaa**ATGGCAAAAGAAGAAGATGAGAAATG) and DJR544(AAAAAAgaatgc **t**GCGGCCGCttaattaa **TCA**AATGAGGTTGCGTGTACG) and cloned the resulting product into pJK590-mut using *Age I* and *Bsm I* to create plasmid pCM14.1. Mutagenesis of the resulting construct yielded plasmids pCM14.2 (A18G), pCM14.3(A18V) and pCM14.4(Q69L).

Transgenes for ectopic DTC-specific CHW-1 expression were generated by co- injection in the *dpy-20(e1282*ts) strain with marker plasmids pPD118.33 (P*myo-2*::*gfp*) 20 ng/μl and pMH86 (*dpy-20(*+)), while P*lag-2*::*chw-1* plasmids were injected at 10 ng/μl. Transgenes were tracked by rescue of the Dpy phenotype and by pharyngeal GFP expression.

The *chw-1* promoter plasmid was constructed by PCR amplification and subcloning of *chw-1* promoter sequences. We attempted to use primers within the F22E12.3 gene upstream of *chw-1*, but resulting constructs caused lethality, preventing isolation of transgenes. Instead we used primers VM59 (forward) TCGAGTATTTCGAACCGTTACTGGTGGAGG (with *Xma I* site) and DJR412 (reverse) TCGAAGTTTTTCCAACTGCC (with *Xma I* site) to amplify promoter sequences not including F22E12.3 coding sequences, and subcloned the resulting product into plasmid pPD95.67, which contains GFP and an *unc-54* 3’UTR (A. Fire, personal communication). This plasmid was injected at 100 ng/μl with co-injection marker plasmid pRF4 (*rol-6(su1006*d)) (80 ng/μl) to make extrachromosomal array *reEx6*, which was then integrated by UV irradiation to form *reIs3*. *reIs3* was outcrossed 5x to the wild type (N2) by following its Rol phenotype, and the resulting strain was used to construct all subsequent strains.

Since *reIs3-*bearing transgenic animals are Rol, it is difficult to see all ventrally located VPCs in one animal. We therefore scored *reIs3* GFP expression in animals grown on *sqt-1*-directed RNAi, which mitigates the *reIs3* Rol phenotype. We independently verified that *sqt-1(RNAi)* did not alter GFP expression patterns.

### 3’RACE

Because the predicted CHW-1 protein lacked typical C-terminal sequences, we performed 3’ RACE experiments to detect all possible 3’ exons. We used forward primers DJR557 in exon 3 (GGAGCTGAATTTTTTGAATGTTCAGC), DJR556 in exon 4 (ATGTTTGACGCAGCAATTTTGGCC) and DJR555 spanning the exon 4/putative 3’UTR boundary (GATTCATCACACGTACTCGCAACC) to amplify from reverse transcriptase-synthesized reverse strand cDNA generated by an Invitrogen 3’RACE kit using total *C*. *elegans* RNA. In each case only one significant 3’RACE band was identified, and sequence analysis indicated that the band corresponded to the Wormbase-predicted gene structure for exon 4, which was also independently validated by cDNA (Wormbase WS226). This predicted gene structure also corresponds to CHW-1 homologs from related nematode species.

## Supporting Information

S1 File An accompanying file contains the list of *C*. *elegans* strains used in this study. (DOCX)

S1 Fig CHW-1 gene structure and sequence in *Caenorhabditis* species. Alignment of predicted CHW-1 proteins from *C*. *elegans* (Ce), *C*. *briggsae* (Cb), and *C*. *remanei* (Cr). Conservative substitutions have gray background and non- conservative substitutions have a white background. The conserved atypical alanine at position 18 is marked with a black arrow.
(TIF)

S2 Fig Detection of *chw-1 (ok697)* by PCR. **A.** *chw-1* gene structure, deletion, and primer location. (B) Gel showing unique band profile of WT (+/+), *chw-1* deletion heterozygotes (*ok697/+)*, and *chw-1* deletion homozygotes (*ok697/ok697)*.
(TIF)

S3 Fig Molecular verification of *lin-18(e620)*. **A.** Representation of PCR products amplified from *lin-18* locus in the wild type and *e620* mutant animals. HpyCH4V restriction sites are denoted with a black triangle **B.** Gel showing unique band profile of WT (+/+), *lin-18(e620)* heterozygotes (*e620/+)*, and *lin-18(e620)* homozygotes (*e620/e620)*.
(TIF)

S4 Fig Detection of *vang-1 (ok1142)* by PCR. **A.** *vang-1* gene structure, deletion, and primer location. **B.** Gel showing unique band profile of WT (+/+), *vang-1(ok1142)* deletion heterozygotes (*ok1142/+)*, and *vang-1(ok1142)* deletion homozygotes (*ok1142/ok1142)*.
(TIF)

S5 Fig The *lin-17*; *lin-18* double mutant does not alter P*_chw-1_*::*gfp* expression. **A.** Fluorescent image of *lin-17(n671)*; *reIs3*;*lin-18(e620)* animal (1000x) at the Pn.px stage, after one cell division. 2°-1°-2° vulval lineages indicated by open white arrows, neighboring non-vulval VPC daughters indicated by solid black arrows. **B.** DIC image of the same animal, with vulval VPC daughters indicated by open black arrows.
(TIF)
