# Supplementary material for: The C. elegans Chp/Wrch Ortholog CHW-1 Contributes to LIN-18/Ryk and LIN-17/Frizzled Signaling in Cell Polarity
Source: PLoS One. 2015 Jul 24;10(7):e0133226. doi: 10.1371/journal.pone.0133226 (PMC4514874; doi:10.1371/journal.pone.0133226)
Supplement: S1 File — (DOCX) [file pone.0133226.s001.docx]

**S1 FILE**

**List of strains used in this study:**

DV2072 *chw-1**(ok697)* 5x outcrossed

DV2574 *chw-1(ok697)* 4x outcrossed

DV2099 *unc-42(e270) sma-1(e30)*

MT458 *unc-20(e112) lon-2(e678)*

DV2050 *cwn-1(ok546)*; *egl-20(n585)*; *chw-1(ok697)*

DV2123 *dpy-20(e1282)*; *reEx21 [*P*_lag-2_::chw-1(Q61L)* *+ dpy-20(+) +* P*_myo-2_::gfp]*

DV2136 *dpy-20(e1282)*; *reEx23 [*P*_lag-2_::gfp +* P*_myo-2_::gfp + dpy-20(+)]*

DV2143 *dpy-20(e1282)*; *reEx25 [*P*_lag-2_::chw-1(A18G) + dpy-20(+) +* P*_myo-2_::gfp]*

DV2145 *dpy-20(e1282)*; *reEx26 [*P*_lag-2_::chw-1(+) + dpy-20(+) +* P*_myo-2_::gfp]*

DV2146 *dpy-20(e1282)*; *reEx27 [*P*_lag-2_::chw-1(+) + dpy-20(+) +* P*_myo-2_::gfp]*

DV2154 *dpy-20(e1282)*; *reEx28 [*P*_lag-2_::chw-1(A18V) + dpy-20(+) +* P*_myo-2_::gfp]*

DV2155 *dpy-20(e1282)*; *reEx29 [*P*_lag-2_::chw-1(A18V) + dpy-20(+) +* P*_myo-2_::gfp]*

MT1306 *lin-17(n671)*

PS1403 *lin-17(sy277)*

DV2492 *lin-17(sy277)*; *chw-1(ok697)*

DV2086 *lin-17(n671)*; *chw-1(ok697)*

CB620 *lin-18(e620)*

MT2129 *lin-18(n1051)*

DV2088 *chw-1(ok697)*; *lin-18(e620)*

DV2489 *chw-1(ok697)*; *lin-18(n1051)*

DV2519 *lin-17(n671)* / *hT2[bli-4(e937)qIs48]*; + / *hT2*; *lin-18(e620)*

DV2516 *lin-17(n671)* / *hT2[bli-4(e937)qIs48]*; + / *hT2*; *chw-1(ok697)*; *lin-18(e620)*

MT1215 *egl-20(n585)*

DV2491 *egl-20(n585)*; *chw-1(ok697)*

DV2528 *lin-17(n671)*; *egl-20(n585)*

DV2527 *egl-20(n585)*; *lin-18(e620)*

DV2529 *lin-17(n671)*; *egl-20(n585)*; *chw-1(ok697)*

DV2526 *egl-20(n585)*; *chw-1(ok687)*; *lin-18(e620)*

NG2615 *cam-1(gm122)*

DV2507 *cam-1(gm122)*; *lin-18(n1051)*

DV2530 *cam-1(gm122)*; *lin-18(e620)*

DV2532 *unc-20(e112) lin-18(e620)*

DV2561 *vang-1(ok1142) lin-18(e620)*

DV2580 *lin-17(n671)*; *cam-1(gm122)*

DV2581 *vang-1(ok1142)*

DV2582 *chw-1(ok697)*; *vang-1(ok1142) lin-18(e620)*

DV2070 *reIs3 [*P*_chw-1_::gfp+rol-6(su1006d)]* 5x outcrossed

DV2080 *lin-17(n671)*; *reIs3 [*P*_chw-1_::gfp+rol-6(su1006*d*)]*

DV2081 *cam-1(gm122)*; *reIs3 [*P*_chw-1_::gfp+rol-6(su1006*d*)]*

DV2082 *reIs3 [*P*_chw-1_::gfp+rol-6(su1006*d*)]*; *bar-1(ga80)*

DV2085 *pry-1(mu38)*; *reIs3 [*P*_chw-1_::gfp+rol-6(su1006*d*)]*

DV2087 *cwn-1(ok546)*; *egl-20(n585)*; *reIs3 [*P*_chw-1_::gfp+rol-6(su1006*d*)*

DV2579 *reIs3 [*P*chw-1::gfp+rol-6(su1006*d*)]*; *lin-18(e620)*

DV2589 *reIs3 [*P*_chw-1_::gfp+rol-6(su1006*d*)]*; *vang-1(ok1142)*

DV2600 *lin-17(n671)* / *hT2 [bli-4(e937)qIs48]*; *reIs3 [P_chw-1_::gfp+rol-6(su1006*d*)]*; *lin-18(e620)*
