# Supplementary figures and images for: The C. elegans Chp/Wrch Ortholog CHW-1 Contributes to LIN-18/Ryk and LIN-17/Frizzled Signaling in Cell Polarity
Source: PLoS One. 2015 Jul 24;10(7):e0133226. doi: 10.1371/journal.pone.0133226 (PMC4514874; doi:10.1371/journal.pone.0133226)

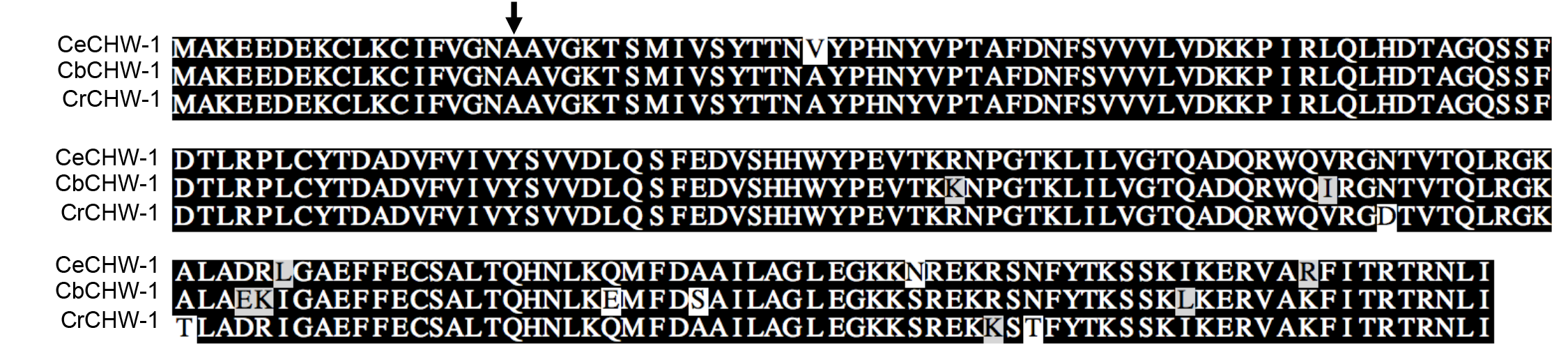

Supplement: S1 Fig — Alignment of predicted CHW-1 proteins from C. elegans (Ce), C. briggsae (Cb), and C. remanei (Cr). Conservative substitutions have gray background and non- conservative substitutions have a white background. The conserved atypical alanine at position 18 is marked with a black arrow. (TIF) [file pone.0133226.s002.tif]

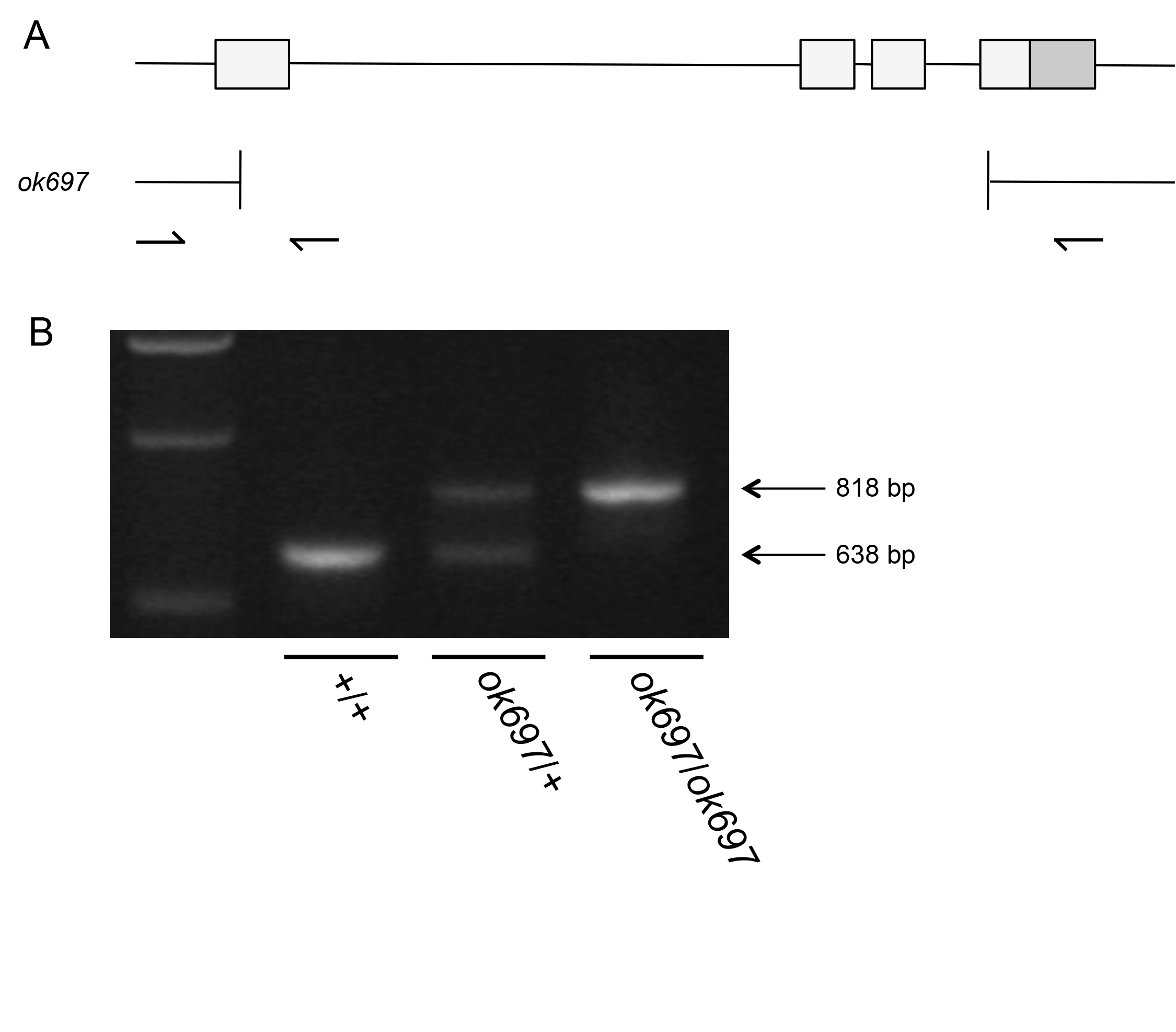

Supplement: S2 Fig — A. chw-1 gene structure, deletion, and primer location. (B) Gel showing unique band profile of WT (+/+), chw-1 deletion heterozygotes (ok697/+), and chw-1 deletion homozygotes (ok697/ok697). (TIF) [file pone.0133226.s003.tif]

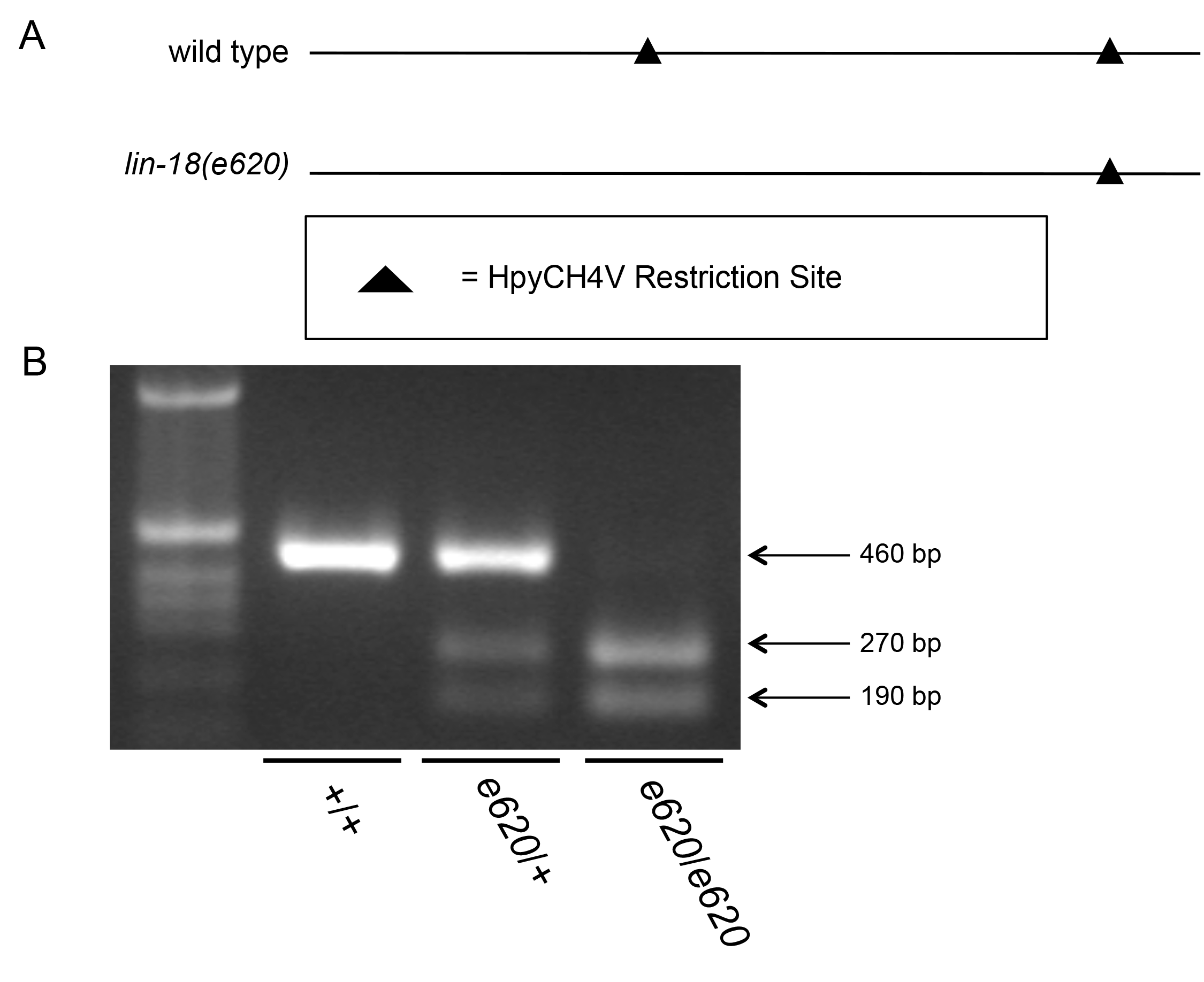

Supplement: S3 Fig — A. Representation of PCR products amplified from lin-18 locus in the wild type and e620 mutant animals. HpyCH4V restriction sites are denoted with a black triangle B. Gel showing unique band profile of WT (+/+), lin-18(e620) heterozygotes (e620/+), and lin-18(e620) homozygotes (e620/e620). (TIF) [file pone.0133226.s004.tif]

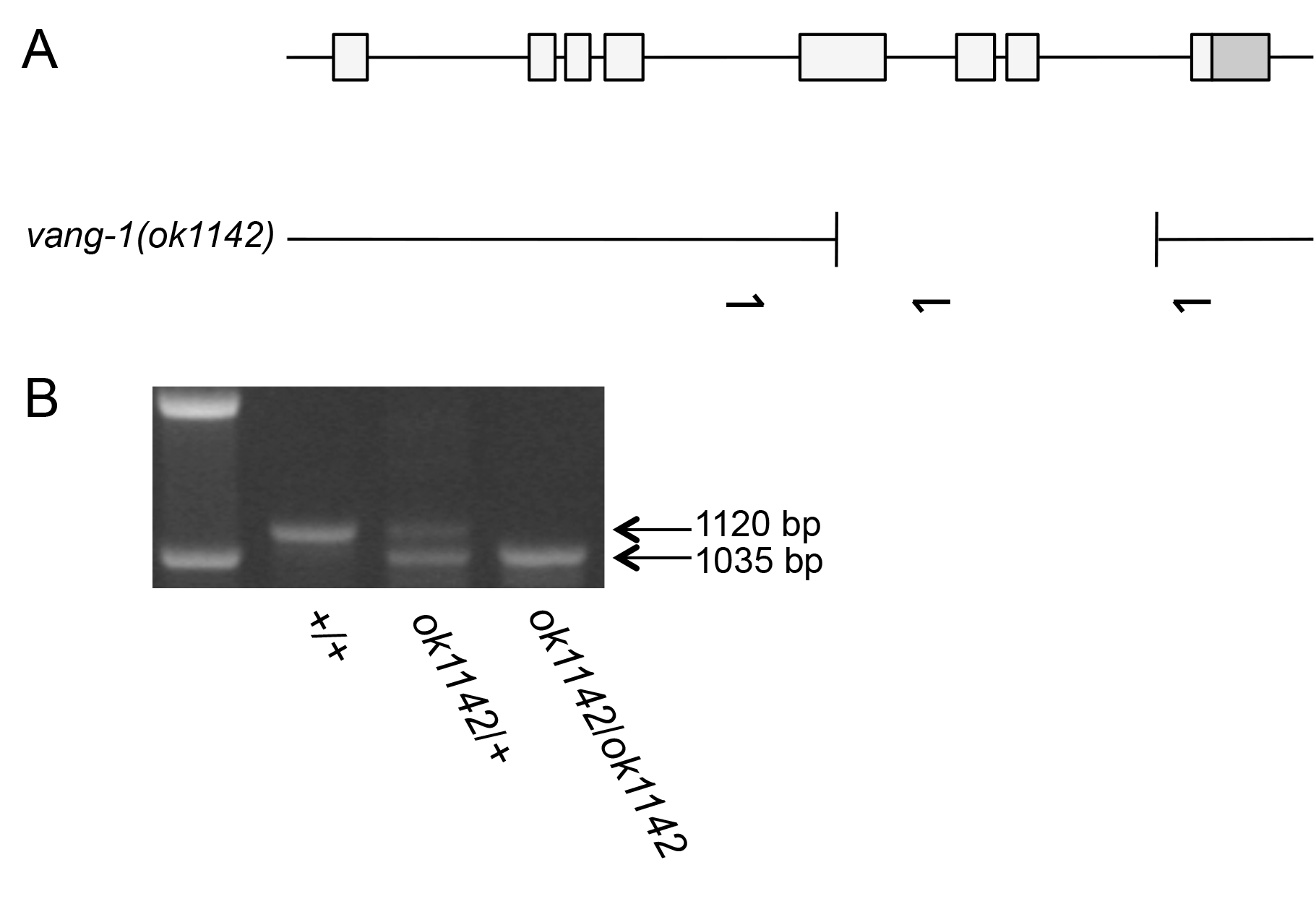

Supplement: S4 Fig — A. vang-1 gene structure, deletion, and primer location. B. Gel showing unique band profile of WT (+/+), vang-1(ok1142) deletion heterozygotes (ok1142/+), and vang-1(ok1142) deletion homozygotes (ok1142/ok1142). (TIF) [file pone.0133226.s005.tif]

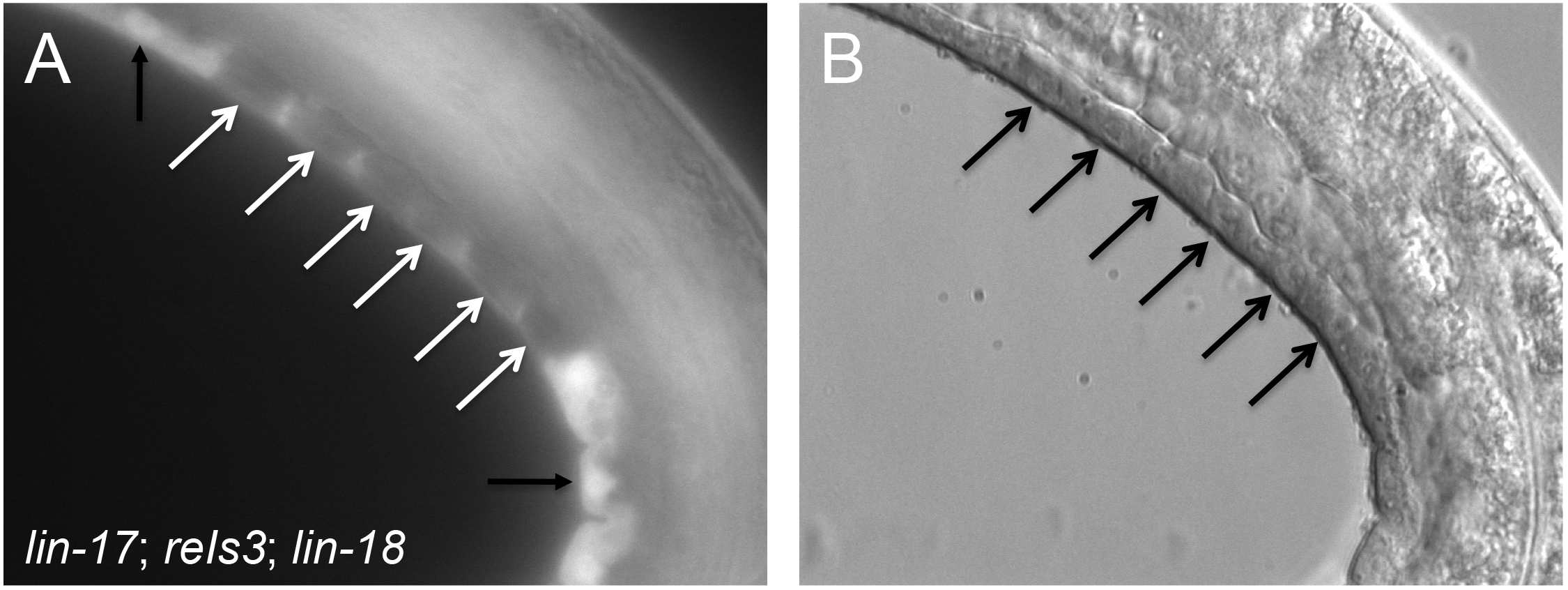

Supplement: S5 Fig — A. Fluorescent image of lin-17(n671); reIs3;lin-18(e620) animal (1000x) at the Pn.px stage, after one cell division. 2°-1°-2° vulval lineages indicated by open white arrows, neighboring non-vulval VPC daughters indicated by solid black arrows. B. DIC image of the same animal, with vulval VPC daughters indicated by open black arrows. (TIF) [file pone.0133226.s006.tif]
